# The Association between Prematurity, Antibiotic Consumption, and Mother-Infant Attachment in the First Year of Life

**DOI:** 10.3390/antibiotics12020309

**Published:** 2023-02-02

**Authors:** Marina Fuertes, Anabela Faria, Joana L. Gonçalves, Sandra Antunes, Francisco Dionisio

**Affiliations:** 1 Centro de Psicologia, University of Porto, 4200-135 Porto, Portugal; 2 Escola Superior de Educação de Lisboa, Instituto Politécnico de Lisboa, 1549-020 Lisboa, Portugal; 3 Hospital de Santo Espírito da Ilha Terceira, 9700-049 Angra do Heroísmo, Azores, Portugal; 4 Universidade Lusíada de Lisboa, 1349-001 Lisboa, Portugal; 5 inED–Centro de Investigação e Inovação em Educação, Escola Superior de Educação, Instituto Politécnico do Porto, 4200-465 Porto, Portugal; 6 Faculdade de Psicologia, Universidade de Lisboa, 1649-013 Lisboa, Portugal; 7 cE3c—Centre for Ecology, Evolution and Environmental Changes & CHANGE—Global Change and Sustai-Nability Institute, Faculdade de Ciências, Universidade de Lisboa, 1749-016 Lisboa, Portugal

**Keywords:** prematurity, antibiotic prescription, mother-infant attachment, maternal sensitivity, strange situation, infant cooperation, microbiota, microbiome, dysbiosis

## Abstract

Antibiotics have individual and public-health drawbacks. Nevertheless, mother-infant attachment quality and maternal sensitivity are associated with antibiotic use. Ambivalent-attached infants are more likely to consume antibiotics than other infants. Conceivably, the emotional over-externalization of ambivalent-attached infants and maternal anxiety when infants are ill raise concerns in healthcare professionals, leading to antibiotic over-prescriptions. However, because infants prematurely born, particularly those with less than 32 weeks of gestation, are under more accurate health vigilance, the impact of infant and maternal behavior on antibiotic prescription may vanish in this sample. To test this hypothesis, we performed a longitudinal study to compare antibiotic use and the quality of mother-infant attachment in three groups: 86 infants born at full-term, 44 moderate-to-late preterm infants (32–36 gestation weeks), and 58 very-to-extreme preterm infants (<32 gestation weeks). Infants’ attachment was observed with the Ainsworth Strange Situation’s experimental paradigm at 12 months of corrected age. Findings indicate that infant attachment strategy is associated with antibiotics uptake, but results vary across samples. The proportion of infants that used antibiotics is highest among ambivalent-attached infants in the full-term sample but highest among avoidant-attached infants in the very-to-extreme premature sample. Moreover, higher infant gestational age and lower maternal sensitivity determine higher antibiotic use.

## 1. Introduction

### 1.1. Antibiotics and Prematurity

Over the last century, sulfonamides, penicillin, and many other antibiotics have selected antibiotic-resistant strains worldwide. The negative consequences of such selection on public health are immense, with about 5 million deaths per year associated with antibiotic-resistant bacteria [1].

On the individual level, antibiotics can also be detrimental. For example, antibiotics prescribed to infants have been associated with the development of obesity, allergies such as asthma, and autoimmune diseases such as type 1 diabetes [2,3,4]. The explanation relies on the antibiotic-induced perturbation of the colonization process of the microbiota, which, without antibiotics, usually undergoes distinct steps and is coherent throughout geographic areas and cultures [3,5]. Preterm infants, particularly very-to-extreme preterm infants, are even more susceptible to antibiotics than full-term infants because their intestines and immune system are more immature at birth [6]. *Enterobacteriaceae*, *Enterococcus*, and other opportunistic pathogenic microorganisms are more abundant, and Bifidobacterium or Bacteroides are less abundant in the stools of preterm infants than in full-term infants [7,8]. Recently, it has been shown that antibiotic use by preterm infants restrained the metabolic pathways required for short-chain fatty acid synthesis, given that butyrate- and acetate-producing genera, such as *Clostridium* and *Blautiai*, were less abundant [9]. This effect of antibiotic exposure is critical because butyrate inhibits the inflammation of colonocytes and enhances epithelial barrier integrity, hence its involvement in the pathogenesis of Crohn’s disease [10].

Moreover, antibiotics can damage brain development [11,12,13]. For example, children treated with antibiotics have an increased risk of severe mental disorders when adults (mainly broad and moderate spectrum of antibiotics) [14,15]. Furthermore, an association between antibiotic use in the first year of life and ensuing neurocognitive effects in childhood, including depression, and anxiety disorders, has been shown [16].

The advantages of antibiotics are so high that the disadvantages of their use can be neglected. With so many negative consequences, including deaths, associated with their usage, it is of utmost importance that all available strategies are used to avoid the overuse of antibiotics. That includes understanding the psychological factors linked with antibiotics prescription [17,18].

### 1.2. Infant Attachment Strategy and Antibiotic Consumption

According to Bowlby’s Attachment Theory (1969), children who maintain proximity to an attachment figure are more likely to receive comfort, care, and protection when feeling distressed or ill and, therefore, more likely to survive to adulthood. This emotional tie, evolutionary selected, relies on a motivational system designed to regulate attachment behavior. Infants who feel pain, fatigue, or sickness obtain proximity and contact using different strategies (secure, insecure-ambivalent, and insecure-avoidant) [19].

Upon conceiving an experimental paradigm (Strange Situation) designed to elicit attachment strategies, Ainsworth et al. (1978) observed three attachment styles (or patterns): secure, insecure-avoidant, and insecure-ambivalent. Infants develop a secure attachment when the caregiver is sensitive to their signals and responds appropriately to their needs. Securely attached infants are easily soothed by the attachment figure when distressed. On the other hand, avoidantly attached infants do not seek contact with the attachment figure when distressed, and their attachment figure is often unavailable during emotional distress. Finally, ambivalently attached infants exhibit inconsistent proximity-seeking and dependent behavior while rejecting the attachment figure. When distressed, they are difficult to soothe and are not comforted by the interaction with their inconsistent attachment figure [20]. Secure attachment is more prevalent in western samples with full-term infants from middle-class households [21]. Beyond caregivers’ behavior, parents’ mental health, maternal education, family social-economic status, and social support from others influence attachment organization [22].

The attachment security is crucial to infant emotional, linguistic, and cognitive development and is associated with health outcomes [23,24,25]. For instance, children with chronic diseases are more likely to present an insecure attachment to their mothers [25,26,27,28]. Furthermore, some studies found that insecure-avoidant and insecure-ambivalent individuals adopt different responses to pain and sickness [25,29]. In fact, while avoidant-attached children and teenagers display high levels of emotional control and low levels of negativity, insecure-ambivalent individuals display low levels of emotionality, facing the same symptoms [24]. Moreover, insecure-ambivalent individuals tend to report more significant symptoms and request more health visits [23,24].

A recent line of research found a link between maternal behavior, infant attachment, and antibiotic use. In a sample mainly composed of infants born full-term, Fuertes et al. [30] found that infants ambivalently attached were more likely to take antibiotics in the first nine months of life than secure and avoidant-attached infants. Another study also found that mothers of infants that take antibiotics are more anxious and less sensitive [31]. It seems that, when ill, ambivalent-attached infants exhibit higher negativity and externalization. In response, attachment figures may become anxious about finding appropriate help care and means to alleviate infants’ pain, expressing their worries to health professionals that may result in preventive antibiotic consumption. However, this effect is attenuated in a sample of infants with low gestational weight, all of them born preterm (both moderate-to-late and very-to-extreme premature infants), probably because some of these infants and their families were supported by early intervention health and developmental services [32].

### 1.3. The Particular Experience of Prematurely Born Infants

Over the last three decades, researchers examined the behavior, development, and relationships of infants born preterm and compared them with those born full-term. Preterm infants have been described as less attentive, organized, and engaged than full-term infants [33,34,35,36]. Their parents were portrayed as more anxious and inconsistent [35,37,38]. Several scholars proposed that when parents struggle to read and support their preterm infant’s behavior (signaling their needs and engaging in positive, congruent, and reciprocal interactions), the transactional interactions between parents and infants may evolve into disturbed relationship patterns. The higher prevalence of attachment insecurity in infants born preterm [39,40] could be a reflex of this disrupted transactional process.

Advances in perinatal and neonatal medical care have significantly increased the survival rates of preterm infants. These care units are highly structured and technological to promote infant survival and provide health care. Thus, much has been done to promote parent-newborn proximity in Neonatal Intensive Care Units (NICUs, e.g., Kangaroo or Newborn Individualized Development Care and Assessment Program methods). Perhaps because of these advances and new family-based interventions, higher rates of secure attachment have been reported for infants born preterm included in these programs [41].

More recently, a body of knowledge has developed to describe infants born very-to-extreme preterm. These infants stay more days in neonatal intensive care units, are at higher life danger, and struggle with more neurobehavioral difficulties, including autonomic, motor and state modulation, arousal regulation, and affective responsiveness, than other infants [42]. Their mothers described the experience of premature labor and the uncertainty of their newborn’s survival as traumatic [43]. At three months of corrected age, infants born very-to-extreme preterm tend to present a negative pattern of regulatory behavior [44], and, at six months of corrected age, a poor behavioral stress modulation [45]. Later, at 12 months of corrected age, infants born very-to-extreme preterm have a higher incidence of insecure attachment compared to other infants [46,47], but results vary across studies [48].

### 1.4. Aims of This Study

The current study has three primary aims. First, we aim to compare the antibiotic use in: (i) infants born full-term; (ii) infants born moderate-to-late preterm; and (iii) infants born very-to-extreme preterm. To the best of our knowledge, this is the first study that compares the three samples concerning antibiotic use. Additionally, we aim to compare these differences according to infant attachment status. On the one hand, we might expect that the proportion of infants that use antibiotics is higher among ambivalent-attached infants than among infants with other attachment patterns in the three infant groups, as we found in previous studies [30,32]. On the other hand, considering that infants born very-to-extreme preterm undergo extra clinical monitoring and health care, the impact of maternal anxiety may fade away in this group.

Finally, we aim to investigate the determinants of antibiotic use from a set of variables: maternal interactive behavior at 3-months during free play interactions; infant interactive behavior at 3-months during free play interactions; and perinatal, family, and social factors.

## 2. Materials and Methods

### 2.1. Participants

Participants were 86 healthy full-term (born with > 37 gestation weeks) infants (35 girls; 51 boys, 47 firstborns), 44 moderate-to-late preterm (born with 32 to 36 gestation weeks) infants (16 girls; 28 boys, 24 firstborns), and 58 very-to-extreme preterm (born with < 31 gestation weeks) infants (28 girls; 30 boys, 38 firstborns). To be eligible to participate in this study, infants had to meet the following inclusion criteria: i) be free of sensory or neuromotor impairments; and ii) be free of severe health conditions (e.g., chronic lung disease), or congenital anomalies at the time of recruitment.

All information regarding the perinatal characteristics of the infants was collected from hospital records from 2017 to 2019. A follow-up of this information was performed at 3, 9, and 12 months of corrected age. Descriptive statistics of the perinatal and demographic characteristics of the participants are provided in Table 1.

### 2.2. Procedures and Measures

All procedures in the present study were conducted according to the ethical guidelines presented in the Declaration of Helsinki, with written informed consent obtained from all individual participants or their legal guardians in the study before any assessment or data collection took place. All procedures were approved by the Ethics Committees of all Health Units and Hospitals involved, as well as by the Portuguese Data Protection Commission.

Participants were recruited two days after birth for the full-term and the moderate-to-late preterm samples, and five to seven days for very-to-extreme preterm samples. At 9 and 12 months postpartum (corrected age), mothers were contacted to schedule a follow-up visit in the laboratory. Mother-infant dyads were videotaped in free play interactions and during the Strange Situation laboratorial procedure [20], when infants were 12 months old of corrected age.

#### 2.2.1. Maternal Sensitivity and Infant Cooperative Behavior

Mother-infant free play interactions were assessed with Child-Adult Relationship Experimental Index (CARE-Index). According to the Care-Index manual [49], maternal sensitivity is any pattern of behavior that pleases the infant, increases the infant’s comfort and attentiveness, and reduces his/her distress and disengagement. The CARE-Index was used to score qualitative dimensions of infant-adult interaction during free play at the 9-month lab visit. The CARE-Index system assesses seven aspects of mother-infant interactive behavior: facial expressions, verbal expressions, position and body contact, affection, turn-taking contingencies, control, and choice of activity. Each adult and infant are evaluated separately in each of these seven dimensions of interactive behavior.

The points for each dimension are added to yield seven scale scores, three adult independent scales, namely Sensitivity, Control, Unresponsiveness and four independent infant scales, namely, Cooperative, Compliant-compulsive, Difficult, and Passive, behaviors [49]. The videotaped free-play interactions were scored by two trained and blind (against the study) coders on the CARE-Index. Intercoder reliability was evaluated by comparing the two coders’ ratings using -intraclass correlation coefficients (ICCs) [50]. The obtained overall average ICCs were 0.79 for full-terms, 0.72 for moderate-to-late preterm, and 0.73 for very-to-extreme preterm. According to manual instructions, each interaction lasted five minutes [49].

#### 2.2.2. Attachment Patterns

Mother-infant attachment patterns were observed using the Strange Situation [20], which is a 21-min laboratory-based observational paradigm involving a sequence of eight brief (3-min) episodes (or less if the infant is distressed) of increasing stress for the infant, including being introduced to an unfamiliar playroom, interacting with an unfamiliar adult stranger, and two mother-infant separations and reunions.

Videotapes of infants’ attachment behavior during the Strange Situation were scored by two trained, reliable coders following the procedures developed by Ainsworth et al. [20]. Infants were classified as either: securely attached (B), insecure-avoidant (A), and insecure-ambivalent (C). Cohen’s kappa coefficients indicated good to excellent intercoder reliability (full-terms *M*κ = 0.79; moderate-to-late preterms *M*κ = 0.72; and very-to-extreme preterms *M*κ = 0.84).

### 2.3. Analytic Plan

In this study, the significance was set at 0.05. Before the aim-oriented analyses, we tested the normality of the variables with Kolmogorov–Smirnov statistics to decide on the tests to perform. Then, we conducted three sets of statistical analyses to address the goals of the current study. First, we used univariate frequency analysis to obtain the distribution of attachment patterns. Second, we performed cross-tabulations to investigate the prevalence of antibiotic use according to the three-prematurity status and attachment patterns. The Fisher-Freeman-Halton test was followed by a z-post hoc analysis to determine group differences in prevalence. Additionally, we used the Bonferroni test correction as a way of proportional reduction in error type I to predict patterns of attachment based on prematurity status. To measure how strongly antibiotic use and attachment patterns are associated (effect size), we calculated Cramér’s V. Third, we performed a binary logistic regression (forward stepwise–Wald) to find the predictors of antibiotic use.

## 3. Results

### 3.1. Associations among Attachment Patterns, Prematurity Status and Antibiotics Uptake

The prevalence of antibiotic use varied across samples: 63.8% of the infants born very-to-extreme preterm (N = 37) took antibiotics during the first 9 months of corrected age, against 40.9% (N = 18) of the infants born moderate-to-late preterm, and 38.4% (N = 33) of infants born at full-term.

Secure attachment prevailed in infants born at full-term (51.2%), but only 38.6% among moderate-to-late preterm infants, and 32.8% among very-to-extreme preterm infants presented the secure-attachment pattern. The ambivalent attachment pattern is more prevalent in moderate-to-late preterm infants (34.1% against 17.4% in full-term infants and 27.6% in very-to-extreme preterm infants), and avoidant attachment is more prevalent in very-to-extreme preterm infants (39.7% against 31.4% in full-term and 27.3% in moderate-to-late preterm infants).

According to Table 2, infant attachment pattern is associated with antibiotic use, but results vary across samples. In the full-term sample, the proportion of ambivalent-attached infants that used antibiotics (93.3%) is higher than among secure (22.7%) or avoidant-attached infants (33.3%). Among moderate-to-late preterm infants, we observed the same tendency (60% of the ambivalent-attached infants but only 23.5% of the securely attached and 41.7% of the avoidant-attached infants consumed antibiotics), but these differences were not statistically significant. In contrast, in very-to-extreme preterm infants, antibiotic use was more common among avoidant infants (82.6%) than among secure (57.9%) and ambivalent infants (43.9%). In addition, we found that most securely attached infants have not consumed antibiotics in the full-term group (77.3%) and the moderate-to-late preterm group (76.5%). According to Fisher-Freeman-Halton test, these associations are significant for full-term preterm infants is 24.144, *p* < 0.001; for moderate-to-late preterm infants is 4.307, *p* = 0.118, for very-to-extreme preterm infants is 6.621, *p* = 0.032. Large to middle-size effects using Carmer’s V were found for the association between attachment pattern and antibiotic use for each birth status group (Cramer’s V = 0.528 for full-term infants; 0.316 for moderate-to-late preterm infants; and 0.337 for very-to-extreme preterm infants).

### 3.2. Factors Associated with Antibiotic Consumption

Infant gestational weight, infant gestational age, maternal behavior, and infant behavior were distinct among infants that used and did not use antibiotics (Table 3). The 100 infants that used antibiotics had a lower gestational age than the 88 infants that did not use antibiotics (*M* = 33.8 weeks, *SD* = 4.55 weeks vs. *M* = 35.6 weeks, *SD* = 3.95 weeks, respectively; *t*(186) = 2.869, *p* < 0.005; Cohen’s *d* = 0.423). The gestational weight of those infants that used antibiotics was lower than that of the infants that did not use antibiotics (*M* = 2.092 kg, *SD* = 0.993 kg vs. *M* = 2.596 kg, *SD* = 0.982 kg; *t*(186) = 3.487, *p* < 0.001; Cohen’s *d* = 0.510]. Moreover, infants that took antibiotics presented more difficulty in free play interactions with their mothers than the infants that did not take antibiotics (*M* = 3.51, *SD* = 3.4 vs. *M* = 2.36, *SD* = 2.8; *t*(186) = 2.516, *p* < 0.01; Cohen’s *d* = 0.372). Furthermore, infants that took antibiotics were less cooperative than infants that did not take antibiotics (*M* = 7.07, *SD* = 2.3 vs. *M* = 8.15, *SD* = 2.38; *t*(186) = 3.227, *p* < 0.001; Cohen’s *d* = 0.471). In turn, mothers of infants that consumed antibiotics were less sensitive and engaged than the mothers of infants that did not take antibiotics (*M* = 6.25, *SD* = 2.3 vs. *M* = 8.16, SD = 2.42; *t*(186) = 3.494, *p* < 0.001; Cohen’s *d* = 0.509).

In infants born at very-to-extreme preterm, the number of stay days in NICU differed according to antibiotic use. Infants that took antibiotics in the first nine months of corrected age remained in NICU in average 68 days against 46 for infants that did not take antibiotics (*M_used antibiotics_* = 67.97, *SD* = 46.09 vs. *M_did not use antibiotics_* = 46.19, SD = 29.79; *t*(56) = 2.182, *p* < 0.05; Cohen’s *d* = 0.531).

### 3.3. Determinants of Antibiotics Use

To find the predictors of antibiotic consumption, we used the significant variables associated with antibiotic use (Table 4) in a binary logistic regression. In the first step, Maternal sensitivity was retained as a predictor, being negatively associated with antibiotic use (β = −0.212, *Wald* = 10.632, *p* = 0.001). The odds ratio for Maternal sensitivity was e^β^ = 0.809, which is lower than 1; similarly, the upper value of the 95% C.I. was lower than 1. Therefore, for one unit increase in Maternal sensitivity, the odds of antibiotic use decreases by approximately 1 − 0.809 = 0.191 = 19.1%. In the second step, Gestational weight (β = −0.445, *Wald* = 8.011, *p* = 0.005) and Maternal sensitivity (β = −0.194, *Wald* = 8.629, *p* = 0.003) were retained as predictors for antibiotic use. The odds ratio for Gestational weight was e^β^ = 0.641, with the upper value of the 95% C.I. below 1 (95% C.I. between 0.471 and 0.872). Therefore, an increase of one kg in the gestational weight (maintaining Maternal sensitivity constant) implies a decrease of 1 − e^−0.445^ ≈ 36% in the odds of taking antibiotics. In this second step, Maternal sensitivity was 0.824, with the upper value of the 95% C.I. below 1. Therefore, for one unit increase in Maternal sensitivity (maintaining Gestational weight constant), the odds of antibiotic use decrease by approximately 17.6%.

## 4. Discussion

Infants exposed to antibiotics in the first two years of life have a significantly higher risk of atopy and the development of metabolic disorders [51]. The risk is even higher among preterm infants, particularly among very-to-extreme preterm infants [6]. Antibiotics often do not distinguish pathogens from commensals, potentially leading to dysbiosis and increasing the odds of infections by enteric bacteria [52,53,54].

Previous research has shown that the proportion of insecure-ambivalent attached infants that took antibiotics in the first nine months of age is significantly higher than among insecure-avoidant and securely attached infants. Moreover, two previous studies have also shown that maternal sensitivity impacts the probability of antibiotic use in the first months of life [30,31]. However, prematurely born infants tend to spend more days in hospitals under close observation and treatments than full-term infants. Also, parents may perceive very premature infants as more fragile and less resistant to infections [55]. Therefore, concerning antibiotic prescription, the very-to-extreme preterm infant’s or caregiver’s psychological factors should be less critical. In this study, we studied three samples (infants born at full-term, moderate-to-late preterm, and very-to-extreme preterm) to understand better the factors involved in antibiotic prescription.

As expected, we found that the proportion of infants that took antibiotics is higher among infants born at very-to-extreme preterm than in the other two groups. Also, as expected, attachment security is more likely in infants born at full-term than in the other two groups [40,44]. Moreover, we did not find significant differences in the attachment insecurity incidence in the other two samples of preterm infants, which suggests prematurity itself is a risk factor for attachment security, as found in past research, e.g. [42].

However, antibiotic use varies across attachment styles by sample. As past studies found with infants born at full term [30], the proportion of infants that used antibiotics in the first nine months is higher in ambivalently attached infants. However, this result is not sustained in the preterm samples. In infants born at very-to-extreme preterm, the prevalence of antibiotic use is greater in infants insecure-avoidant attached (82.6%), compared with secure (57.9%) and insecure-ambivalent attached (43.9%) infants. One possible explanation is that separation from parents, invasive and painful health care in NICU, and prolonged hospital stays may promote the development of avoidant attachment patterns. Others found infant avoidant attached are more likely to stay longer in NICU [56].

This difference across samples of infants born at full term versus very-to-extreme preterm is very intriguing. In prior research, studies were performed with participants with different prematurity statuses together. For instance, in a study mainly conducted with infants born at full term but also around 20% of preterm infants [30], the prevalence of antibiotic use in ambivalent-attached infants was explained by over-externalizing of emotions among these infants, which may worry their parents when ill, and thereby parents request antibiotic prescriptions or over-report their infant symptoms to health professionals. However, the effect is much weaker in a sample of infants with low birthweight (composed only of prematurely born infants). In our study, comparing the three samples, we found that avoidant-attached infants were the ones that took more antibiotics in very-to-extreme preterm infants.

One possible explanation for these results relies on medical support and vigilance offered to infants born at very-to-extreme preterm. In Portugal, teams of specialized medical doctors and nurses follow up on these infants in the first year of life in regular health consultations, usually in public hospitals or family health units. Health vigilance of these infants is more frequent than that of infants born at full term or moderate-to-late preterm, which can supersede the effect of “parental anxiety” on antibiotics prescription. It is possible that parents of infants born at very-to-extreme preterm feel more assured of attending health appointments regularly, obtaining more reliable medical information, and establishing trusting relationships with health professionals. Also, these infants are prioritized in hospitals and emergencies, and parents are likely to receive medical aid when feeling necessary. Thus, the excessive externalization of emotions in ambivalent children may not affect antibiotic prescription to these infants born at very-to-extreme preterm as it happens in infants born at full term.

There is another intriguing observation concerning infants born at very-to-extreme preterm: the proportion of insecure-avoidant attached infants that used antibiotics was 33.3% among infants born at full term and 41.7% among infants born at moderate-to-late preterm, but 82.6% among infants born at very-to-extreme preterm. In our study, the latter commonly remain for more days in NICU and undergo more intrusive and painful life-support than other infants. Although presently NICUs promote baby-skin contact of newborns with parents through the Kangaroo method, we speculate that infants born at very-to-extreme preterm tend to develop an avoidant attachment resulting from (i) the initial separation from parents; (ii) invasive and painful treatments during their prolonged stays in hospitals; and (iii) feeling of lack of protection from parents. This should be further investigated.

This study suggests that infants with specific attachment patterns are at higher risk of antibiotics overuse. A recent qualitative study in England indicated that mothers tend to overvalue antimicrobials, asking them even for viral infections. Mothers said they feel more comfortable if their children take antibiotics as a preventive treatment or when necessary [57]. Corroborating that parents’ behavior and prematurity have a critical impact on antibiotics use, we found that maternal sensitivity and gestational age are determinants of this consumption for all three samples together.

Since antibiotic use could favor the start of neurodevelopmental disorders [13], we may ask: can antibiotics impact infant’s attachment pattern? If they can, we may add changes in infant-mother attachment patterns to a long list of behavioral effects of antibiotics [13]. However, a comparison of studies suggests that the answer is no. Indeed, while the present study shows that the prevalence of antibiotic use is greater in insecure-avoidant attached infants born at very-to-extreme preterm, a previous study has shown that antibiotic use is greater in insecure-ambivalent attached infants born at full term [30]. Therefore, if antibiotics impact the attachment pattern, the effect is very different between very-to-extreme preterm and full-term infants. Future studies should address this conundrum.

It is crucial to consider the limitations and strengths of this study to evaluate the results. First, the sample size is relatively small, reducing the statistical power of the analyses regarding the distribution of attachment patterns. Also, in future studies, it is important to consider parental stress and mothers’ perceptions of their infant’s health status and fragility.

This study’s longitudinal, prospective design, together with the use of direct observations of mother-infant interaction at nine months and attachment at 12 months (corrected age), are strengths of this research. With the study’s aims in mind, we evaluated the biological and demographic factors as potential covariates. Thus, we believe the present findings add to a growing body of knowledge about attachment and antibiotics use in infants born preterm and full-term [17,30,31,32].

## 5. Conclusions

Adding to a body of research that underlies the need to work collaboratively with parents, we found that maternal sensitivity and gestational age are determinants of antibiotic use. Moreover, antibiotics use is more likely in ambivalent-attached infants for full-term and moderate-to-late preterm infants, while the proportion of avoidant-attached infants that used antibiotics is highest in the very-to-extreme preterm infants’ sample. Therefore, to prevent the misuse of antibiotics in pediatrics, professionals must work collaboratively with parents and use distinct strategies according to infant prematurity status. Health general practitioners working with early intervention professionals (a multidisciplinary family-based intervention to support children under six years old), who perform home visits, need to explain the use of antibiotics to families, help them to read children’s health symptoms, and how to access to rapid health responses in case of illness [17,18].

## Figures and Tables

**Table 1:** Samples’ characteristics.

Demographics	Full-Term			Moderate-to-Late Preterm			Very-to-Extreme Preterm		
	*M*	*SD*	Min-Max	*M*	*SD*	Min-Max	*M*	*SD*	Min-Max
Gestational Age (weeks)	38.8	1.08	37.0–41.3	33.86	1.7	32.0–36.5	29.5	1.88	23.5–31.6
Gestational weight (kg)	3.286	0.465	2.000–4.635	2.116	0.507	1.301–3.160	1.171	0.304	0.500–1.995
Apgar at first minute	8.75	0.79	4–10	8.16	1.36	3–10	6.69	2.23	1–9
Apgar at fifth minute	9.76	0.73	4–10	9.32	0.83	7–10	8.55	1.72	2–10
Maternal age (years)	30.08	5.01	19–43	30.52	5.7	18–43	33.16	6.03	18–46
Maternal education (years)	13.12	3.68	7–23	14.32	3.67	8–22	13.14	3.98	3–23
Number of siblings	1.21	0.75	0–4	0.52	0.93	0–5	0.72	0.89	0–4
Number of days in NICU	0	0	0–0	17.04	11.62	0–33	60.09	42.07	7–205

**Table 2:** Frequency of attachment patterns at 12 months of corrected age according to prematurity status and antibiotic use in the first nine months of corrected age.

			Attachment Classification 12 Months ^1^		
			Avoidant	Secure	Ambivalent
		Antibiotic			
Prematurity status	Full-term	Yes	9 (33.3%, −0.7) ^a^	10 (22.7, −3.1%) ^a^	14 (93.3%, 4.8) ^b^
		No	18 (66.7%, 0.7) ^a^	34(77.3%, 3.1) ^a^	1 (6.7%, −4.8) ^b^
	Moderate-to-late preterm	Yes	5 (41.7%, −0.1) ^a^	4 (23.5%, −1.9) ^a^	9(60%, 1.9) ^a^
		No	7 (58.3%, −0.1) ^a^	13(76.5%, 1.9) ^a^	6(40%, −1.9) ^a^
	Very-to-extreme preterm	Yes	19 (82.6%, 2.4) ^a^	11 (57.9%,−0.7) ^a,b^	7(43.9%, −2.0) ^b^
		No	4 (17.4%, −2.4) ^a^	8 (42.1%, 0.7) ^a,b^	9(56.3%, 2.0) ^b^
	Total	Yes	33 (53.2%, 1.2) ^b^	25 (31.3%, −3.7) ^a^	30 (65.2%, 2.9) ^b^
		No	29 (46.8%, −1.2) ^b^	55 (68.8%, 3.7) ^a^	16 (34.8%, −2.9) ^b^

^1^ The Fisher-Freeman-Halton test for infants born at full-term is 24.144, *p* < 0.001, Cramer’s V = 0.528; for moderate-to-late preterm is 4.307, *p* = 0.118, Cramer’s V = 0.316, for very-to-extreme preterm is 6.621, *p* = 0.032, Cramer’s V = 0.337; and for Total is 15.135, *p* < 0.001, Cramer’s V = 0.283. Each superscript letter (a, b) denotes a subset of Attachment at 12-months categories whose column proportions do not differ significantly from each other; *p* < 0.05 (column proportions test with Bonferroni adjustment). For all cases, Cramér’s V values suggest that there is an intermediate or large association between antibiotic use and attachment patterns. In the case of the moderate-to-late preterm infants, despite the intermediate effect size (Cramér’s V = 0.316), the *p*-value for these infants is non-significant or marginally significant (*p* = 0.118), possibly due to the small sample size of this group of infants (N = 44).

**Table 3:** T-test for mean differences of infant and maternal factors according to antibiotics usage.

Infant and Maternal Variables	Took Antibiotics			Did Not Take Antibiotics					
	*N*	*M*	*SD*	*N*	*M*	*SD*	*t*	*p*	Cohen’s *d*
Maternal sensitivity	88	6.95	2.3	100	8.16	2.42	3.494	<0.001	0.509
Maternal control	88	4.2	3.34	100	3.82	3.14	0.81	0.42	0.119
Maternal unresponsivity	88	2.76	3.18	100	2.04	2.38	1.742	0.08	0.259
Infant cooperative behavior	88	7.07	2.3	100	8.15	2.38	3.227	0.001	0.471
Infant compulsive behavior	88	2.32	2.28	100	1.92	3.12	0.849	0.40	0.125
Infant difficult behavior	88	3.51	3.4	100	2.36	2.8	2.516	0.01	0.372
Infant passive behavior	88	1.28	1.89	100	1.61	2.2	1.076	0.28	0.156
Gestational age	88	33.8	4.55	100	35.6	3.95	2.869	0.005	0.423
Gestational weight	88	2.092	0.993	100	2.596	0.982	3.487	<0.001	0.510
Apgar 1st minute	88	7.81	1.86	99	8.11	1.64	1.185	0.24	0.175
Apgar 5th minute	88	9.24	0.86	99	9.32	1.19	0.561	0.58	0.081
Maternal age	88	31.63	5.94	100	30.7	5.35	1.116	0.27	0.164
Maternal education	88	13.25	4.03	100	13.54	3.57	0.519	0.60	0.076
Number of siblings	88	0.86	0.85	100	0.93	0.92	0.514	0.61	0.075

**Table 4:** Summary of binary logistic regression analyses predicting antibiotic use.

		β	S.E.	Wald	df	*p*	Exp(β)	95% C.I. for Exp(β)	
								Lower	Upper
**DV: Antibiotic use**									
1st step	Maternal sensitivity	−0.212	0.065	10.632	1	0.001	0.809	0.712	0.919
	Constant	1.491	0.509	8.583	1	0.003	4.443		
2nd step	Gestational weight	−0.445	0.157	8.011	1	0.005	0.641	0.471	0.872
	Maternal sensitivity	−0.194	0.066	8.629	1	0.003	0.824	0.724	0.938
	Constant	2.394	0.620	14.906	1	<0.001	10.954		

## Data Availability

The data presented in this study are available on request from the corresponding author. The data are not publicly available due to data confidentiality and to protect participants’ privacy.
